# Fault tree analysis-adapted knowledge structuring: a case study of sustainable international security cooperation

**DOI:** 10.3389/frai.2026.1723198

**Published:** 2026-02-06

**Authors:** Yudai Wada, Koki Ijuin, Chiaki Oshiyama, Takuichi Nishimura

**Affiliations:** School of Knowledge Science, Japan Advanced Institute of Science and Technology, Nomi, Japan

**Keywords:** knowledge structuring, fault tree analysis, CHARM, knowledge graph, tacit knowledge, sustainable knowledge transfer, international security cooperation, R&D and procurement

## Abstract

Sustainable knowledge transfer in Japan’s international security cooperation for research and development (R&D) and procurement is challenging due to institutional and security constraints. Critical know-how is often tacit and dispersed among experts; continuity is undermined by frequent personnel rotations, temporal–spatial gaps between projects, and institutional and cultural differences across international partners, leading to knowledge loss. In this context, traditional on-the-job training is difficult to sustain, making durable knowledge transfer difficult to achieve. To overcome this problem, this study proposes a knowledge engineering method that externalizes practitioner expertise. Procedure-based knowledge (what/how) and purpose-based knowledge (why: purposes and decision rules) are structured as two auditable linked, machine-interpretable graphs—the procedure- and purpose-based knowledge graphs. This method uses the Convincing Human Action Rationalized Model (CHARM) as a notation for knowledge structuring. To articulate implicit causal reasoning, we integrate Fault Tree Analysis (FTA) as a qualitative, deductive elicitation and articulation notation. Starting from observed outcomes (“top events”), FTA deductively elicits avoidance purposes and candidate actions. These purpose-action pairs are then recorded under Reference Cases (RC) and embedded as RC-tagged links in the two graphs with FTA-derived annotations, thereby refining causal logic and facilitating knowledge externalization. We empirically assess the method’s effectiveness through qualitative and quantitative analyses of data from semi-structured interviews, a facilitated workshop, and FTA-guided follow-up interviews. These activities increased both the volume and granularity of externalized knowledge, yielding 42 RCs and 133 case-attached actions as provenance-bearing purpose-action units. Our approach yields reusable, machine-interpretable assets for human-AI collaboration and may support continuity during constrained handovers, which may help mitigate repeated errors and improve negotiation preparedness. These findings suggest that the FTA-adapted CHARM approach can foster more sustainable knowledge transfer for Japan’s international security cooperation.

## 1 Introduction

Japan’s 2022 National Security Strategy prioritizes international security cooperation (Government of Japan, 2022). Within this policy framework, research and development (R&D) and procurement frameworks are central to strengthening partnerships, interoperability, and risk- and cost-sharing (Ministry of Defense, 2024). Despite this institutional momentum, the sustainable transfer of critical knowledge remains challenging for four systemic reasons (Wada et al., 2025). First, the absence of formal manuals limits the accumulation of explicit knowledge. Second, the flow of tacit, domestically anchored knowledge is governed by strict regulatory and security constraints. Third, personnel rotations occur every two to three years, combined with five- to six-year gaps between projects, which cause temporal–spatial discontinuities and repeated procedural errors. Fourth, Japan’s knowledge transfer system lacks the structural integration found in the United States, wherein institutionalized personnel exchanges occur across the military, defense industry, think tanks, and Congress (McGann, 2016; Coyne and Goodman, 2022; Gehlhaus et al., 2023; Wang, 2024). Collectively, these cultural and organizational factors constrain knowledge flow (Wada et al., 2025).

To address these issues, we adopt a knowledge engineering approach. Prior work on machine-interpretable knowledge structuring has leveraged knowledge representation and semantic technologies to improve consistency and reusability. We use an activity model called the Convincing Human Action Rationalized Model (CHARM) as an ontology-aligned notation that describes task actions in a purpose-oriented, hierarchical goal-means manner. CHARM decomposes each action into subordinate actions required to achieve its purpose, thereby structuring the relationship between actions and purposes (Nishimura et al., 2013). Building on this approach, we externalize knowledge into two complementary types for preservation and transfer: procedure-based knowledge (what/how actions are performed) and purpose-based knowledge (the why: purposes and decision rules). We represent these as two separate but linked knowledge graphs (KGs): the procedure- and purpose-based knowledge graphs. Linking multiple purpose nodes to each action node enables comprehensive externalization and reduces omissions (Ijuin et al., 2022; Ijuin and Nishimura, 2023).

Within this framework, CHARM serves as the dedicated notation for structuring this knowledge by organizing actions and purposes through task analysis and hierarchical goal-means decomposition (Nishimura et al., 2013; Iino et al., 2020a; Ijuin and Nishimura, 2023). To reveal implicit causal reasoning that conventional interviews often miss, we employ Fault Tree Analysis (FTA)—a method typically used for safety and reliability analysis—as a qualitative elicitation and structuring notation. Starting from a defined “top event,” FTA deductively decomposes it into contributory events (Lee et al., 1985; NASA, 2002; IEC 61025, 2006; Ruijters and Stoelinga, 2015). We integrate FTA outputs into the CHARM-based representations and introduce a Reference Case (RC) field to record FTA-derived instances, linking them consistently to procedures and purposes. In summary, CHARM provides the structured canvas, while FTA supplies the deductive scaffold to clarify multi-purpose causal interactions and facilitate the externalization of tacit knowledge.

Prior research establishes that CHARM structures action-purpose relations and FTA clarifies causal logic. However, neither conventional interviews nor on-the-job training alone provides a deductive, auditable route to externalize latent purposes into machine-interpretable units. This study examines how sustainable knowledge transfer can be achieved under temporal–spatial discontinuities and institutional constraints in international R&D and procurement. We therefore couple FTA-guided backward reasoning with RC-anchored purpose-action links embedded in the two graphs.

This study develops a knowledge engineering approach with three primary contributions. Methodologically, we integrate FTA as both an analysis method and an articulation notation and introduce an RC procedure that uses observed outcomes to deductively elicit avoidance purposes and candidate actions; these are encoded as purpose-action pairs annotated with FTA events, adding a deductive causal scaffold that ensures traceability and makes tacit rationales explicit, thereby supporting auditability and reproducibility. Theoretically, this operationalizes a deductive route for rendering tacit rationales into machine-interpretable purpose-action units within a CHARM-based representation. Practically, we implement the resulting artifacts in two complementary, linked knowledge graphs—the procedure- and purpose-based knowledge graphs—to make the many-to-many relationships between purposes and actions explicit, queryable, and reusable under national-level constraints.

The remainder of this paper is structured as follows: Section 2 reviews the relevant literature on tacit and explicit knowledge, knowledge structuring (including CHARM), KGs, and FTA. Section 3 presents our proposed FTA-adapted elicitation workflow, the corresponding CHARM-consistent notational additions, and describes the empirical implementation in a Foreign Military Sales (FMS) case (research design, participants, integrated procedure, and data management and rigor safeguards). Section 4 reports the results, including the knowledge externalization counts, the resulting procedure- and purpose-based knowledge graphs, a quantitative analysis of case-attached actions and qualitative validation feedback. Section 5 synthesizes the methodological, theoretical, and practical implications, discusses positioning relative to existing approaches, discusses limitations, and outlines directions for future work. Finally, Section 6 concludes the paper by summarizing our findings.

## 2 Related work

### 2.1 Knowledge transfer frameworks

#### 2.1.1 Explicit and tacit knowledge

In today’s competitive global environment, the strategic role of knowledge has intensified, becoming a fundamental driver of innovation, resilience, and sustainable development. Foundational work by Polanyi (1966), Nonaka (1994), and Davenport and Prusak (1998) established that knowledge creation, transfer, and utilization are integral to organizational learning and long-term competitiveness. Moreover, Davenport and Prusak (1998) emphasized that knowledge transfer, including spontaneous and unstructured flows, is a vital part of organizational life and performance. Effective knowledge governance—encompassing its creation, dissemination, and adaptation—directly impacts organizational learning, technological capabilities, and national competitiveness.

A core distinction in knowledge science is that between explicit and tacit knowledge (Polanyi, 1966; Nonaka, 1994). Explicit knowledge refers to content that can be systematically documented and shared, such as in operational manuals, blueprints, or analytical formulas. By contrast, tacit knowledge is deeply rooted in action, experience, values, and context. It is often acquired through socialization rather than formal instruction, which makes it difficult to verbalize or codify (Nonaka and Takeuchi, 1995). As Polanyi (1966) emphasized, this knowledge has a personal, practice-grounded character. Therefore, the central challenge in knowledge management is to effectively create, capture, and transfer tacit knowledge, as it constitutes a substantial portion of expert practice. This requires mechanisms to externalize tacit knowledge into structured, shareable forms while preserving its original context. Methodologies such as ontology-based modeling and explicitation interviews are key to this transition, particularly in domains that require fine-grained contextualization (Chergui et al., 2020).

#### 2.1.2 Knowledge transfer in international R&D and procurement

Japan’s international security cooperation in R&D and procurement involves multi-actor coordination across domestic and international organizations, regulatory constraints, and temporal–spatial discontinuities caused by personnel rotations and multi-year gaps between projects (Sakaki and Maslow, 2020; Ogi, 2025; Wada et al., 2025). These arrangements require sustained expertise and robust information management under international negotiation and cost-control pressures. Representative frameworks include international joint R&D, Direct Commercial Sales (DCS), and the Foreign Military Sales (FMS) program (Defense Security Cooperation University, 2022; Defense Security Cooperation Agency, 2025).

Within these frameworks, knowledge transfer must traverse intercultural, institutional, and regulatory boundaries, often under stringent information security constraints. Key challenges include legal discrepancies, heterogeneous security protocols, and uneven baseline knowledge across stakeholders, which can disrupt both tacit and explicit exchanges (Defense Security Cooperation University, 2022; Migone et al., 2023; Ministry of Defense, 2024; Defense Security Cooperation Agency, 2025). Organizational and cultural conditions can suppress the uptake of externally sourced knowledge, a dynamic observed in both skilled return migration (Wang, 2015) and R&D and procurement for international security cooperation.

A recurrent obstacle is “stickiness,” that is, the difficulty of moving knowledge in a usable form, which arises from causal ambiguity, low absorptive capacity, trust gaps, and arduous relationships (von Hippel, 1994; Szulanski, 1996, 2000). Recent evidence shows that knowledge barriers can mediate the relationship between the knowledge source and transfer performance, an effect that can be moderated by political skill (Lee et al., 2022). Thus, sustained knowledge transfer depends on both institutional mechanisms and interpersonal facilitation. In academic settings, for instance, tacit knowledge exchange is enabled by a combination of individual factors, organizational structure, and knowledge management strategies (Alves and Pinheiro, 2022).

Operationally, certain roles and practices can mitigate these challenges. For example, “bridge managers” help reconcile divergent organizational logics by translating goals, aligning expectations, coordinating procedures, and mediating political and cultural divides in cross-border R&D (Uchihira et al., 2017; Chalarak, 2021; Chalarak et al., 2021, 2023). Complementing this role, structured project cases and internalization workshops offer repeatable vehicles to convert experience into reusable management knowledge, which helps offset the long cycles and high uncertainty typical of R&D (Uchihira, 2010, 2015; Uchihira et al., 2012). As these transfers occur in policy-intensive environments, combining strategic foresight with sustainability transitions frameworks can support the co-creation of adaptable solutions under complex conditions (Matti et al., 2025). Taken together, these perspectives motivate a structured, layered approach that makes tacit purposes explicit and reusable—an agenda increasingly implemented with hybrid human-artificial intelligence (AI) methods.

#### 2.1.3 Hybrid human-AI models

Advancements in AI and natural language processing (NLP) have produced powerful new methods for knowledge elicitation, particularly in domains where critical expertise is tacit and difficult to externalize. By codifying and reusing knowledge that would otherwise remain undocumented, AI-enhanced systems can reduce knowledge attrition in complex environments (Zaoui Seghroucheni et al., 2025). This has created opportunities for hybrid human-AI systems that combine computational efficiency with human judgment and contextual awareness. For example, in engineering design, AI-based extraction successfully complements human expertise, demonstrating the value of this collaborative approach (Krahe et al., 2021).

Furthermore, security research has verified the effectiveness of this hybrid approach. A recent analysis of the Russia–Ukraine conflict used a mixed-methods pipeline integrating AI-assisted semantic analysis with expert validation to decode manipulative narratives, demonstrating a viable model for rigorous human-AI partnerships in high-stakes environments (Paziuk et al., 2025). When applied to international security cooperation, such hybrid models can mitigate knowledge “stickiness.” Specifically, by balancing computational efficiency with human interpretability and oversight, they help clarify causal logic and augment the absorptive capacity of recipients. This, in turn, strengthens knowledge transfer and decision-making in dynamic environments with significant institutional and security constraints.

### 2.2 Knowledge structuring and the SECI process

In knowledge engineering, researchers use knowledge representation and semantic technologies to build machine-interpretable knowledge structures, enhancing knowledge consistency and reusability. These approaches often use ontologies to define notations that describe task actions in a purpose-oriented, hierarchical structure. One such notation is CHARM, which describes actions using a goal-means, purpose-oriented structure. CHARM decomposes each action into the sequence of subordinate actions required to achieve its purpose, thereby making the relationships between actions and their goals explicit (Nishimura et al., 2013).

Furthermore, structured representation has gained momentum in domains with high procedural complexity. In CHARM, lower-level action nodes are performed to achieve higher-level ones, and nodes can carry attributes such as conditions and actors (Nishimura et al., 2013). When combined with domain ontologies, CHARM-based descriptions become machine-interpretable, enabling the consistent encoding of tasks (Nishimura et al., 2013, 2017). Prior applications in fields such as caregiving and nursing have shown that this structuring helps surface practitioner rationales often missing from manuals. Subsequent work has extended this applicability to education, construction, and manufacturing, demonstrating both pedagogical and operational utility (Nishimura et al., 2018).

Building on these foundations, recent studies have introduced explicit purpose representations that link multiple purposes to a single action, which better mirrors expert practice and supports reuse across tasks (Iino et al., 2020b; Ijuin and Nishimura, 2023). In addition, this line of work uses workshop-based practices to structure work procedures, elicit the purposes behind each action, link the two knowledge types, and review tasks to deepen understanding (Ijuin et al., 2022; Ijuin and Nishimura, 2023).

This methodological approach aligns with the SECI process, a theoretical lens that describes the dynamics between tacit and explicit knowledge through four phases: Socialization, Externalization, Combination, and Internalization (Nonaka and Takeuchi, 1995). A related concept, Ba, highlights the relational context that enables this knowledge co-creation (Nonaka et al., 2000). In this study, representing procedure- and purpose-based knowledge as two linked graphs corresponds to the externalization and combination phases, allowing procedures and purposes to be articulated, connected, and recombined for long-term organizational learning. Accordingly, this study employs CHARM strictly as a notation for structuring actions and purposes, while using FTA as an analysis method to make implicit causal reasoning explicit.

### 2.3 Knowledge graphs (KGs)

A KG structures facts, concepts, and relations for reuse across applications (Hogan et al., 2021; Fan and Chen, 2024). In practice, KGs support a range of functions, including efficient search via pattern matching (Song et al., 2018), question-answering through weakly supervised multi-hop reasoning (Bi et al., 2023), aiding inference under uncertainty by learning confidence-aware embeddings of facts and relationships (Yang et al., 2022). KG-based models can also provide interpretable decision support in complex domains such as international security cooperation, where interdependent factors must be handled systematically (Gao et al., 2023).

In this study, KGs serve as the organizational infrastructure for knowledge structuring and transfer. We encode entities, activities, and relations in a machine-interpretable format with explicit provenance to facilitate validation, reuse, and the combination of rule-based reasoning with data-driven updates (Noy et al., 2019; Hogan et al., 2021). Concretely, procedure- and purpose-based knowledge are represented in two separate but linked KGs—procedure- and purpose-based (Ijuin et al., 2022; Ijuin and Nishimura, 2023). This design enables procedures and their underlying purposes to be connected, audited, and reused across different tasks and sites.

### 2.4 Fault tree analysis (FTA)

FTA is a versatile analytical tool used to evaluate the reliability and safety of complex systems (Lee et al., 1985). It is a deductive, top-down method that models how conditions and events lead to a defined, undesired “top event.” The causal structure is represented as a logic tree that supports both qualitative and quantitative analyses (NASA, 2002; IEC 61025, 2006; Ruijters and Stoelinga, 2015). Although best known for safety-related domains, FTA can also address availability and maintainability. Moreover, it has a success-oriented counterpart, known as Success Tree Analysis (STA) (IEC 61025, 2006).

Methodologically, FTA starts from the top event and traces backward to identify single or combined causes, complementing inductive approaches that reason forward from causes to their effects. The two approaches are complementary within safety, risk, and reliability engineering. As a decision-support tool, FTA excels at: (i) clarifying causal logic, (ii) prioritizing contributors, (iii) guiding preventive design, and (iv) supporting performance monitoring (NASA, 2002).

In this study, we adopt FTA as both an analysis method and articulation notation. An unmet purpose is treated as the top event, contributory sub-goals as intermediate events, and concrete actions as basic events. The resulting FTA outputs are encoded as annotations and attached to purpose and action nodes in our CHARM-based representations. This makes implicit causal reasoning explicit and preserves traceability from purposes to actions. For scope and terminology, we draw on the classic survey literature (Lee et al., 1985; NASA, 2002; IEC 61025, 2006).

### 2.5 Research gap

Despite substantial progress in knowledge modeling, the effectiveness of existing approaches is limited by three persistent gaps when applied to the practical R&D and procurement challenges of Japan’s international security cooperation.

First, although FTA is a well-established method in safety and reliability engineering, its current guidance is predominantly oriented toward technical failure logic (NASA, 2002; IEC 61025, 2006; Ruijters and Stoelinga, 2015). Even though prior work has integrated FTA with machine-interpretable formalisms and human actions, a notable gap remains in the literature: to the best of our knowledge, no peer-reviewed studies have provided an auditable procedure for modeling human cognitive purposes and purpose-action failures as primary elements within FTA. Furthermore, none have directly evaluated FTA’s effectiveness as an explicit mechanism for externalizing tacit expert rationales into reusable structures. This distinction matters because CHARM is a structuring notation rather than an analysis method (Nishimura et al., 2013; Iino et al., 2020b). Table 1 summarizes the respective roles of CHARM and FTA, and contrasts them with the proposed FTA-adapted CHARM approach.

**Table 1:** Comparison of CHARM, FTA, and the FTA-adapted CHARM approach.

Dimension	CHARM	FTA	FTA-adapted CHARM (this study)
Primary aim	Structuring actions and purposes	Deductive analysis of causal factors	Externalize and link purposes to procedures with a deductive scaffold
Unit of representation	Action–purpose in task hierarchy	Events, gates (AND/OR), top event	Action, purpose, event; RC as provenance tag
Elicitation directionality	Mostly inductive/task-driven	Deductive (from top event)	Hybrid: deductive FTA guiding elicitation + CHARM structuring
Why–how linkage	Assigning purposes is possible	Not assumed	Many-to-many purpose-action links across two KGs
Auditability/traceability	Limited provenance	Model logic traceable; human intent implicit	End-to-end traceability via RC and purpose-action links
Typical outputs	Hierarchical task models	Fault trees, cut sets	RC-tagged purpose-action pairs embedded in two KGs

Note: RC = Reference Case (defined in Section 3).

Second, purposes and intentional logic are often deeply embedded and pre-reflective, making them difficult to verbalize (Wada et al., 2025)—a challenge consistent with the SECI process’s internalization phase (Nonaka and Takeuchi, 1995). While existing CHARM-based structuring effectively captures procedures and links multiple purposes to a single action (Nishimura et al., 2013, 2018; Ijuin et al., 2022; Ijuin and Nishimura, 2023), our empirical work identifies some significant limitations of this approach. Specifically, many purposes remain excessively tacit and context-dependent to articulate in conventional interviews, and existing elicitation protocols lack disciplined procedures to reason backward from outcomes to reconstruct causal logic. Consequently, a method that structures not only observable actions but also their underlying rationale—including contextual, cognitive, and situational factors—in a machine-interpretable and reusable format consistent with CHARM-based representations is required.

Third, international security cooperation in R&D and procurement remains underexplored from a knowledge structuring perspective at the national level. A range of constraints—including security classifications, institutional heterogeneity, personnel rotations, complex information management, and temporal–spatial discontinuities—impede direct handovers and knowledge continuity (Ministry of Defense, 2024; Wada et al., 2025). In addition, these challenges obscure causal pathways and rationales, underscoring the need for a deductive framework, such as FTA, to externalize and stabilize knowledge under these demanding institutional conditions.

This study addresses these gaps by developing a knowledge engineering approach. This approach provides a scalable and auditable foundation operable in human-AI settings for sustainable knowledge transfer in Japan’s international security cooperation for R&D and procurement.

## 3 Methodology

This section describes the methodology in two parts. First, we develop an FTA-adapted knowledge structuring mechanism that externalizes purpose-based knowledge and formalizes FTA-derived annotations as backward-compatible extensions to CHARM. Second, we describe the empirical research design and implementation of the method in an FMS procurement case, including data collection, the integrated elicitation procedure (interviews, workshop, and FTA-guided follow-ups), and the validation and traceability protocol. This separation clarifies the boundary between method development and empirical implementation, improving rigor and reproducibility.

### 3.1 Method development: FTA-adapted knowledge structuring mechanism

Prior structuring work using CHARM—an action notation grounded in goal-means and purpose-oriented modeling—has effectively captured procedural hierarchies (Nishimura et al., 2013, 2018). Building on this, recent studies have represented knowledge in two separate but linked KGs—procedure- and purpose-based—allowing multiple purposes to be linked to a single action (Ijuin et al., 2022; Ijuin and Nishimura, 2023). However, eliciting the latent intentional logic that underpins expert actions remains challenging.

To better articulate this logic, we integrate FTA as a complementary analysis method and knowledge-articulation notation. FTA is a deductive, top-down technique that starts from an observed top event and decomposes it into logically related intermediate and basic events. The method is standardized in IEC 61025 (2006), with authoritative guidance provided in the NASA Fault Tree Handbook (NASA, 2002), and a consolidated methodological review (Ruijters and Stoelinga, 2015). In this study, we adapt FTA qualitatively as an elicitation scaffold—rather than for probabilistic quantification—to externalize purpose-based knowledge that CHARM alone struggles to surface. Accordingly, the role of FTA in this study is methodological (elicitation and articulation), not probabilistic risk estimation.

**Figure 1:**
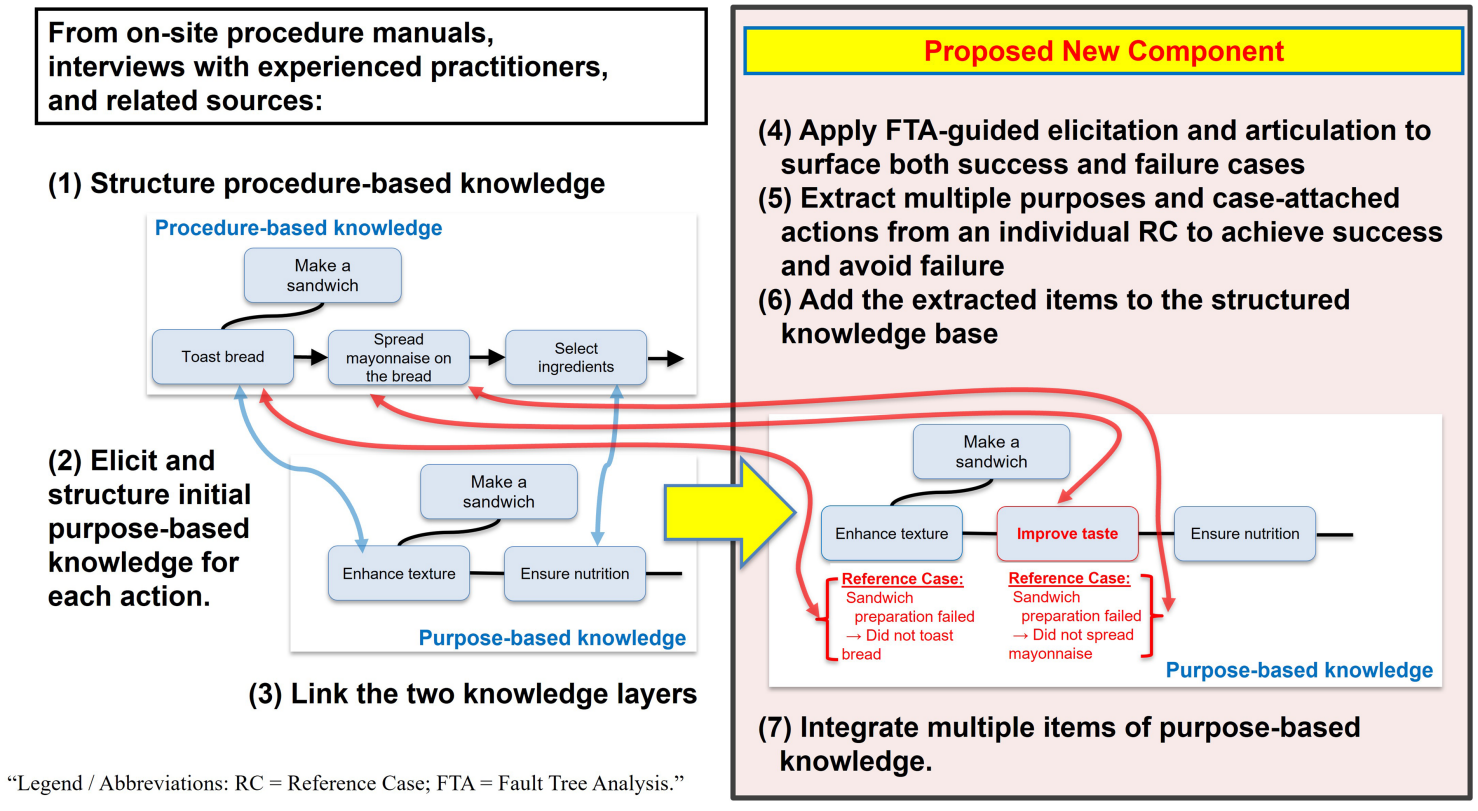
Proposed FTA-adapted knowledge structuring methodology.

The proposed workflow (depicted in Figure 1) delivers two main outcomes: (i) externalization of purpose-based knowledge and (ii) two consistently linked graphs with RC annotations on purpose and action nodes, which enable cross-case retrieval and gap identification. An RC is defined as an individual, well-specified episode of success or failure used for structured elicitation and analysis.

The inputs for this procedure are drawn from on-site manuals, interviews with experienced practitioners, and related sources. For transparency, we express the mechanism as an explicit sequence from (i) procedure capture to (ii) RC-based deductive elicitation guided by FTA to (iii) graph-level integration and reuse. The workflow proceeds as follows (Figure 1):

1. Structure procedure-based knowledge
2. Elicit and structure initial purpose-based knowledge for each action
3. Link the two knowledge layers
4. Apply FTA-guided elicitation and articulation to surface both success and failure cases
5. Extract multiple purposes and case-attached actions from an individual RC to achieve success and avoid failure
6. Add the extracted items to the structured knowledge base
7. Integrate multiple items of purpose-based knowledge

Steps (1)–(3) establish a baseline representation and linkage. Step (4) then serves as a deductive scaffold that uses FTA to explicate RC-level success/failure rationales and refine and clarify the purpose articulations elicited in Step (2). Steps (5)–(7) operationalize RC-based extraction and integration for reuse by converting an individual RC into multiple purpose-action units, registering them in the knowledge base, and consolidating overlapping purpose-based knowledge across cases.

Figure 1 uses a purely illustrative, non-domain example (sandwich preparation) to show how procedure-based knowledge (e.g., “Make a sandwich → Toast bread → Spread mayonnaise on the bread → Select ingredients”) is linked to purpose-based knowledge (e.g., “Enhance texture → Improve taste → Ensure nutrition”) and how case-linked failures (e.g., “Sandwich preparation failed → Did not toast bread → Did not spread mayonnaise”) are captured.

A key advantage of FTA is its support for reasoning backward from outcomes. Beginning with concrete episodes of success or failure, participants can systematically trace contributory purposes and actions, making tacit rationales explicit and reducing causal ambiguity. Success episodes can be handled by reformulating the top event as a failure to achieve the desired outcome and then analyzing its negation. In our adaptation, an unmet-purpose state corresponds to the top event, contributory unmet-purpose states are modeled as intermediate events, and actions or conditions serve as basic events. This process enables experts to reconstruct their decision logic and record it in a machine-interpretable, reusable format consistent with CHARM-based representations.

Accordingly, we introduce FTA-guided follow-up interviews as a structured elicitation step. The elicited causal structures are then mapped onto CHARM’s purpose-action hierarchy, with FTA outputs encoded as annotations and registered as RC entries. This representation is designed to ensure that the procedure- and purpose-based KGs are consistently linked and auditable, which enables cross-case retrieval and targeted gap identification (Nishimura et al., 2013; Ijuin et al., 2022; Ijuin and Nishimura, 2023). The specific notational additions that operationalize this mapping within CHARM are described in Section 3.2.

### 3.2 Notational extensions to CHARM

This section specifies the notational extensions designed to implement the FTA-adapted method. The goal is to encode case-derived causal logic (FTA events and gate relations) and provenance (RC tags) inside CHARM-consistent purpose-action structures, while preserving CHARM’s goal-means semantics and enabling auditability, reuse, and cross-case retrieval.

#### 3.2.1 Overview of the CHARM notation

**Figure 2:**
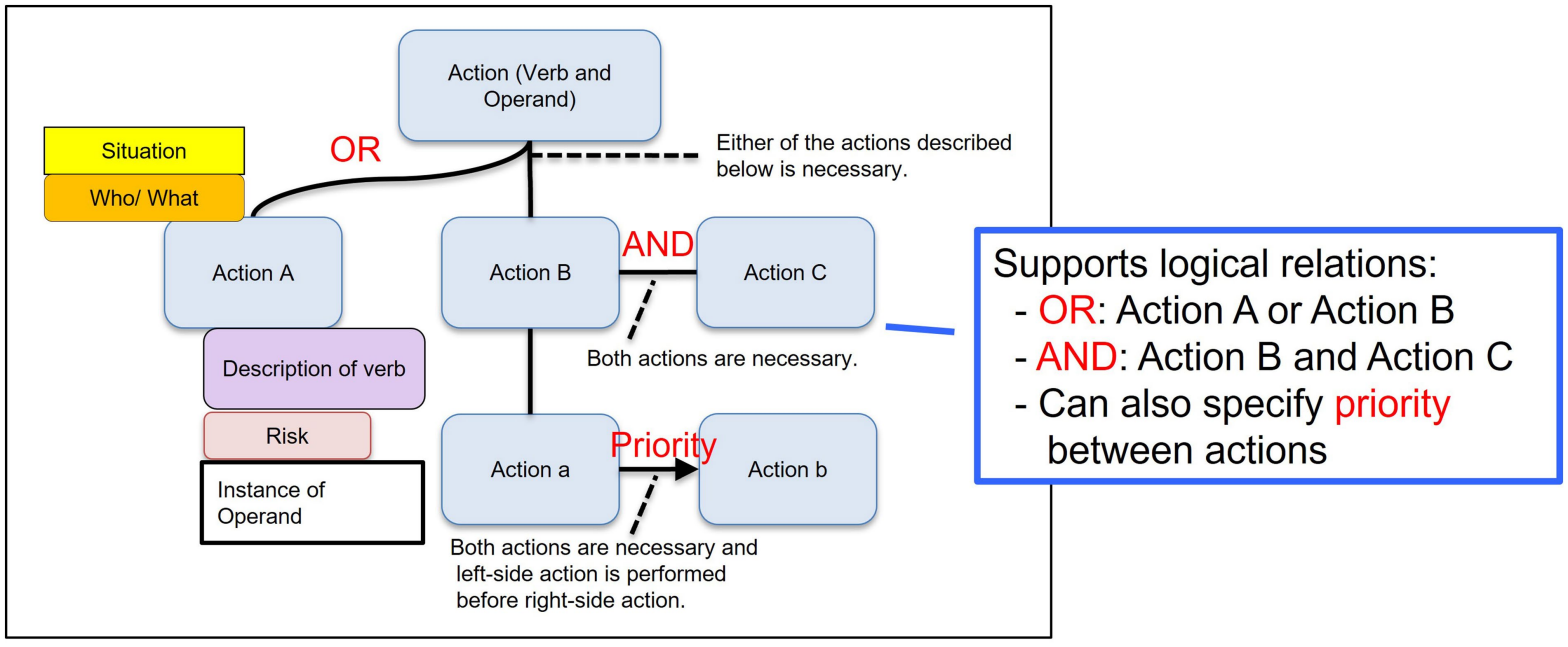
Basic structure of the CHARM notation.

CHARM is a goal-means notation that decomposes a higher-level action into the lower-level actions required to achieve it (Nishimura et al., 2013). As illustrated in Figure 2, each action is represented in a simple “verb + noun (operand)” format and can be accompanied by contextual cards, such as Situation and Who/What (actor/role), with optional Verb description, Risk, and Operand instance. The notation also supports basic logical relations among actions, such as OR (alternative actions), AND (jointly required actions), and Priority (a required execution order).

#### 3.2.2 Substantive FTA-derived extensions to CHARM

We introduce two notational additions to encode FTA outputs within CHARM while keeping the goal-means and purpose-action semantics intact.

##### 3.2.2.1 Reference case (RC) notation

Starting from a seed case, each observed outcome (or “top event”) is analyzed with FTA to deductively elicit the corresponding avoidance purposes and candidate actions. These purpose-action pairs are then encoded and bound to an RC, which externalizes tacit rationales and stabilizes case-specific knowledge for reuse. In practice, presenting a concrete RC alongside a purpose-action pair provides an immediately understandable example that improves practitioner comprehension during authoring and review. These FTA-derived annotations materially strengthen the case-based validation of purposes and improve traceability across the two graphs without altering CHARM’s underlying meaning.

##### 3.2.2.2 Case-attached actions and linking

For each observed outcome in a single case, we extract multiple purpose-action pairs and formalize the actions as case-attached actions. These pairs are then embedded and linked within the purpose-based KG—explicitly recording the RC-derived relationships—and cross-linked to the corresponding actions in the procedure-based KG where actions are anchored. This process ensures traceability and makes purposes queryable and reusable, which is critical under the institutional constraints and temporal–spatial discontinuities typical in international security cooperation in R&D and procurement.

Collectively, these extensions embed concrete cases into explicit, reusable purpose-action structures and provide machine-interpretable provenance through RC tags and FTA-derived annotations, all while keeping CHARM’s semantics intact. When registered in the structured knowledge base, these are treated as complete knowledge units, consistent with the method described in Section 3.1.

### 3.3 Empirical research design and case context

To empirically operationalize and evaluate the method developed in Sections 3.1–3.2, we conducted a qualitative multi-method case study in FMS procurement. The design adopts a structured methodology using a combination of semi-structured interviews, a facilitated expert workshop, and FTA-guided follow-up interviews. The method was implemented in the context of FMS procurement, wherein these difficulties are particularly salient.

#### 3.3.1 Case study: foreign military sales (FMS) procurement

FMS is a government-to-government (G-to-G) program through which a seller nation provides defense articles, services, and training to allied and partner nations (Defense Security Cooperation University, 2022). The seller’s government contracts and procures items on the partner’s behalf through its own defense acquisition system. In the United States, the program is administered by the Department of Defense via the Defense Security Cooperation Agency and is governed by the Security Assistance Management Manual (Defense Security Cooperation Agency, 2025).

Our field data indicate that FMS exemplifies the core difficulties addressed by this study. In this domain, both procedure-based knowledge and, particularly, purpose-based knowledge remain underarticulated. The process requires coordination among multiple domestic and international actors under strict constraints and relies on high-stakes information management across long, fragmented life cycles. These features make FMS complex and increase the risk of errors, establishing it as a priority for improvement. Therefore, we selected FMS as the primary case to analyze knowledge transfer bottlenecks and test whether a structured, machine-interpretable representation of knowledge can sustain continuity and enable human-AI applications.

#### 3.3.2 Data collection, participants, and ethics

We employed a qualitative multi-method design that combined semi-structured one-on-one interviews with a facilitated expert workshop and targeted FTA-guided follow-up interviews for causal elicitation. This arrangement is consistent with established practices in qualitative social research, which uses individual interviews to elicit personal perspectives and small focus groups (our workshop) to surface shared meanings (Morgan, 1997; Parent et al., 2000; Kvale and Brinkmann, 2009). To complement the qualitative findings, we also conducted descriptive quantitative analyses, such as calculating the counts and distributions of case-attached actions. An overview of the data-collection process is presented in Table 2.

**Table 2:** Overview of the data-collection process.

Component	Implementation in this study
Design	Qualitative multi-method design: Semi-structured one-on-one interviews; Facilitated expert workshop (small focus group); FTA-guided follow-up interviews (with thematic coding supported by kNeXaR-based CHARM structuring).
Participants	Sample size across all phases: *n* = 30 (participants). Current/former practitioners (government and private sector; domestic and international).
Instruments	Interview guide: task sequences, decision points, information dependencies, success/failure cases.
Recording protocol	With prior permission from each participant: in-person interviews recorded on a dedicated digital voice recorder; online interviews recorded using the conferencing platform’s built-in recording function.
Procedures	Interviews: approximately 60 min each. Workshop: approximately 120 min. FTA-guided follow-up interviews: approximately 60 min each.

Note: kNeXaR = kNowledge eXplication augmenteR (defined in Section 3).

All procedures were approved by the Knowledge Science Ethics Council at the Japan Advanced Institute of Science and Technology. All participants provided written informed consent before their involvement. Interviews were audio-recorded only with prior permission, and the resulting files were transcribed, de-identified, and stored on encrypted drives with restricted access.

### 3.4 Empirical implementation: integrated knowledge structuring procedure

**Figure 3:**
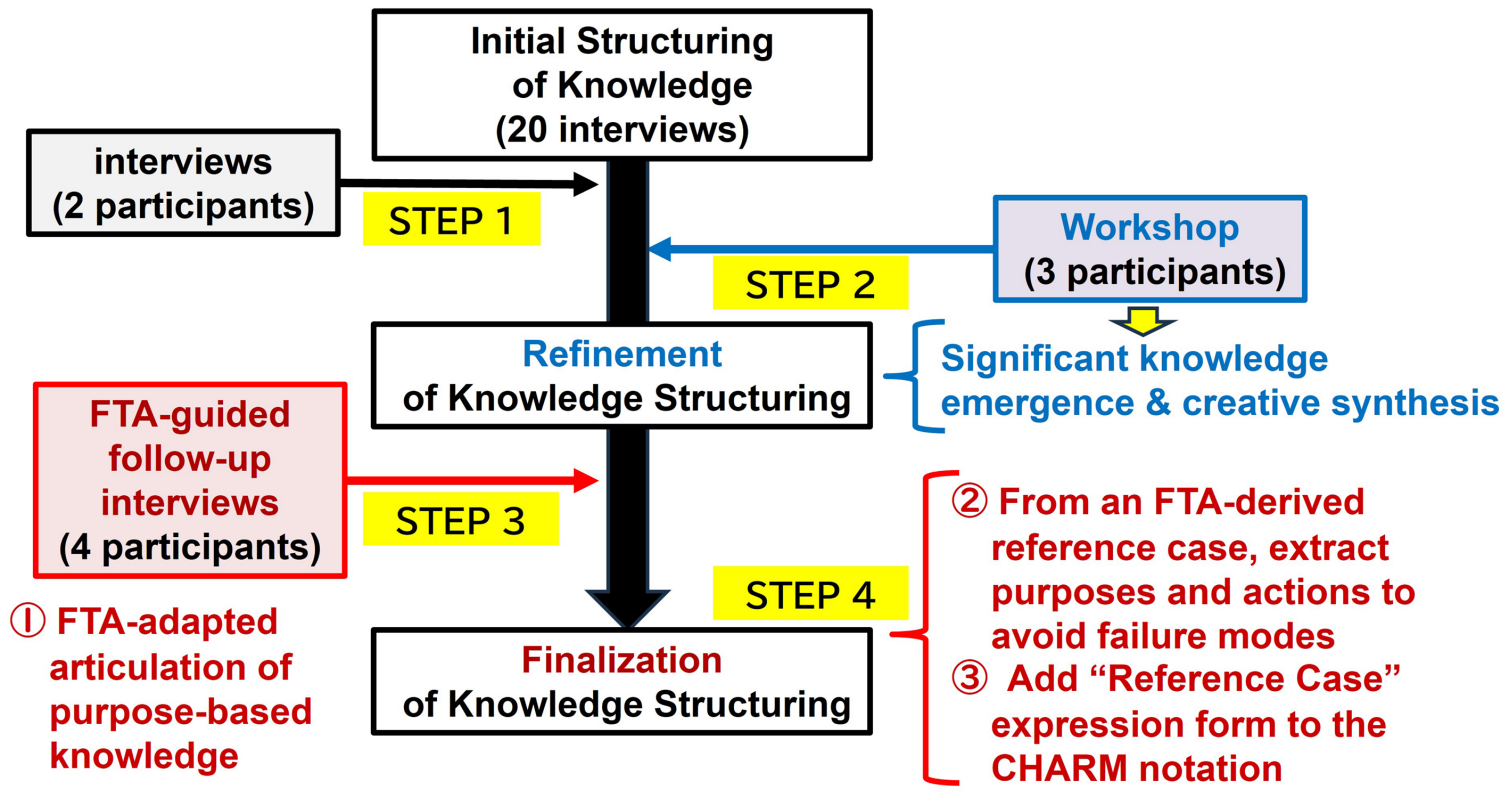
Integrated process for knowledge structuring, interview, workshop, and FTA.

Each phase produces auditable artifacts (node IDs, provenance links, and RC tags) that support cross-phase integration and later quantitative summaries. We implemented the FTA-adapted CHARM approach to externalize and link two layers of knowledge—procedure- and purpose-based—as paired graphs with clear traceability between purposes and actions. Figure 3 illustrates this integrated process, which combines interviews, a workshop, and FTA-guided follow-up interviews.

#### Step 1: Initial structuring from interviews

We first coded data from an initial corpus of 20 interviews to create a draft CHARM representation. Each action node (verb + operand) was assigned a unique node ID with provenance links to the source data and contextual information. This draft was then refined through follow-up interviews with two participants.

#### Step 2: Workshop-based refinement

A facilitated workshop with three participants (one facilitator and two experts) was conducted to harmonize terminology and validate process flows that were difficult to confirm in individual interviews. Materials from the interviews and workshop were thematically coded, and candidate purposes and actions were iteratively mapped into the CHARM structure.

#### Step 3: FTA-guided follow-up interviews

Using the refined structure, four participants engaged in FTA-guided follow-up interviews. Starting from observed outcomes, they used backward reasoning to reconstruct causal chains and articulate the underlying purpose-based knowledge, formalizing each episode as an RC. From each RC, we extracted the purposes and actions intended to avoid failure modes and registered them as RC-tagged purpose-action pairs (Figure 3: red items ①–③).

#### Step 4: Integration and finalization

The results were consolidated into procedure- and purpose-based KGs with explicit bidirectional links for goal-means alignment. RCs were attached to relevant nodes, a change log was maintained, and internal consistency checks (including the absence of orphan nodes, consistent terminology, and traceable sources) were performed to prepare the artifacts for reuse and cross-case retrieval.

### 3.5 Data management and rigor

This subsection clarifies the operational procedures used to transform field data drawn from past materials, interviews, workshop outputs, and FTA-guided follow-ups into RC-tagged, CHARM-consistent knowledge graph entries. To ensure reproducibility under comparable constraints, we describe the data-handling pipeline, coding strategy, definitions of knowledge units, and validation steps.

#### 3.5.1 Data handling and preprocessing

All audio recordings were transcribed verbatim and then de-identified by removing names, organizational/unit identifiers, and time-sensitive operational details. Each transcript was assigned a unique source document ID (e.g., Procedure-xx, Purpose-xx, RC-xx) and segmented into short meaning units, which were then normalized into a verb + noun (operand) format to support traceable structuring (Nishimura et al., 2013; Iino et al., 2020b; Ijuin and Nishimura, 2023). Workshop artifacts (e.g., whiteboard notes, worksheets, and sticky-note annotations) were digitized and managed by decomposing them into knowledge units with structured fields (i.e., verb + noun (operand), optional verb and noun details, identified issues/problems, and case-based categories). A structured audit trail was maintained, consisting of (i) raw transcripts (securely managed with restricted access), (ii) de-identified working transcripts, (iii) structured excerpts, (iv) intermediate representations (CHARM inputs and analytic descriptions using case-specific FTA trees), and (v) finalized RC-tagged structured knowledge artifacts and knowledge graphs.

#### 3.5.2 Interview guide structure and elicitation targets

To ensure procedural consistency, we conducted the semi-structured interviews using the already-developed procedure- and purpose-based knowledge graphs and applied an interview guide that maps directly to the two-layer representation and RC-anchored elicitation. The guide comprised the following modules:

1. Procedure capture (procedure-based knowledge): Eliciting step sequences, branching conditions, exceptions, and verification points. Action statements were normalized into CHARM-consistent expressions in a “verb + noun (operand)” format (Nishimura et al., 2013; Iino et al., 2020b).
2. Case anchoring (RC candidates): Eliciting concrete success/failure episodes, observable outcomes, and contextual conditions. These elements were used to define top events and to populate RC metadata (e.g., RC type: success/failure; top event label) for subsequent FTA-guided elicitation.
3. Rationale capture (purpose-based knowledge): Eliciting decision points, avoidance purposes, and decision rules that explain why a given action is needed in a specific case, as well as cues used by practitioners (e.g., timing, relevant counterparts, and content) (Ijuin et al., 2022). For example, in a de-identified excerpt, one participant stated: “When a time-critical procurement document is delayed, we first confirm the latest status and bottlenecks with relevant counterparts, then coordinate internally to decide what can be accelerated and what requires formal approval. Based on that, we negotiate the next steps and timelines.”

The workshop followed the same modules but placed greater emphasis on shared meaning-making, reconciliation of divergent accounts, and facilitation techniques designed to promote co-creation and emergent collaboration (Sawyer, 2006, 2014, 2018). This enabled joint generation of baseline procedure and purpose articulations and RC candidates.

In this study, coding was operationalized as CHARM-consistent structured entry in kNeXaR (kNowledge eXplication augmenteR). We then structured and registered the de-identified transcripts and workshop materials in kNeXaR, a knowledge structuring support system, using CHARM notation (Iino et al., 2020b). During entry, we selected ontology terms to standardize labels and support term control. Structuring proceeded in three passes: (i) procedure-focused entry to identify and consolidate action candidates (including operands, ordering, and branching conditions) and normalize them into CHARM-consistent “verb + noun (operand)” expressions; (ii) purpose-focused entry to capture purposes (desired states/avoidance aims) and decision rules (conditions governing action selection, prioritization, or omission); and (iii) RC anchoring to bind the resulting purpose-action items to RC identifiers and observable outcomes for subsequent FTA-guided refinement.

For reproducibility, we defined three minimal “knowledge units” in the structured knowledge base: (i) action units, (ii) purpose-action units, and (iii) case-attached actions. Each unit references the unique source document ID assigned in Section 3.5.1, and we added an RC identifier notation to preserve provenance. When multiple accounts referred to the same action under different contexts, we represented variability through multiple links, decision-rule attributes and contextual-condition attributes, and/or distinct RC tags.

#### 3.5.3 Construction and validation of qualitative FTA trees

FTA was applied qualitatively to each RC as a deductive elicitation scaffold and as an intermediate representation linking field narratives to CHARM-consistent entries. The procedure was standardized as follows.

1. Define the RC and top event: For each RC, we fixed the episode boundary (actors, timing, location, and observable outcome) and defined the top event. For failure RCs, the top event was the observed undesired outcome; starting from it, we iteratively elicited contributory factors and the intended “should-be” state to surface fine-grained purpose-based knowledge. For success RCs, we anchored the analysis in the achieved desired outcome and its enabling factors, and additionally analyzed its negation by reformulating a complementary top event as a failure-to-achieve-the-desired-outcome. This ensured that purpose-action items were elicited in an FTA form comparable across success and failure RCs.
2. Elicit and normalize events: In FTA-guided follow-up sessions, participants decomposed the top event into contributory event statements and then into candidate actions/conditions. Contributory events were recorded as concise event phrases and treated as contributory unmet-purpose states at the purpose layer, while actions/conditions were treated as basic events and mapped to candidate actions at the procedure layer.
3. Transform FTA outputs into RC-tagged, CHARM-consistent entries: We transformed intermediate qualitative FTA representations into newly defined RC-tagged knowledge graph entries during kNeXaR-based structuring. We recorded qualitative AND/OR gates (without probabilistic quantification) in each case-specific tree (NASA, 2002; IEC 61025, 2006; Ruijters and Stoelinga, 2015). A practitioner familiar with the RC reviewed the tree to confirm plausibility and interpretation. We registered basic events (actions/conditions) as CHARM-consistent action nodes. We registered contributory events as purpose-layer nodes. We encoded conditional statements as decision-rule attributes and contextual-condition attributes. We recorded resulting purpose-action links as RC-grounded case-attached actions. Each entry preserved provenance by referencing the unique source document ID (Section 3.5.1) and the RC identifier notation.
4. Gap identification and prioritization of follow-ups: After integration, we conducted targeted gap checks (e.g., actions without purpose links, purposes without case-attached actions, or causal claims lacking gate logic) and prioritized additional FTA-guided follow-ups under the same protocol.

## 4 Results

### 4.1 Effectiveness of knowledge elicitation methods

This section reports on the knowledge structuring process, focusing on the volume and type of knowledge externalized through different methods. Table 3 reports the month-by-phase distribution of externalized knowledge units, classified into procedure- and purpose-based knowledge, where each increment is attributed to the elicitation phase conducted in that month (interviews in January–February, workshop in March, and FTA-guided follow-ups in April–May).

**Table 3:** Monthly count of externalized knowledge units by type (FMS case).

Knowledge type	Initial total	January 2025	February 2025	March 2025	April 2025			May 2025	Total
		Interview	Interview	Workshop	FTA follow-up			FTA follow-up	
					#1	#2	#3		
Procedure-based knowledge	405	16	17	72	17	12	18	19	576
Purpose-based knowledge	307	10	11	26	31	43	53	39	520

Note: Values are per-phase increments; Total = Initial total + sum of increments.

Knowledge externalization was evaluated by measuring the content articulated during semi-structured interviews, a facilitated workshop, and FTA-guided follow-up interviews. Building on prior research demonstrating the difficulty of externalizing the tacit rationale behind actions (Nishimura et al., 2017; Iino et al., 2020b; Ijuin and Nishimura, 2023; Wada et al., 2025), we distinguished between procedure-based knowledge (tasks, sequences, and conditions) and purpose-based knowledge (the goals and decision rules that guide practice). The data in Table 3 reflect the accumulation of both knowledge types, clarifying how each elicitation technique revealed different layers of expertise as externalized units.

The results highlight the differential output profiles across methods. The facilitated workshop in March resulted in a substantial increase in procedure-based knowledge, yielding more than twice the volume of the preceding interviews. This suggests that collective refinement in a workshop setting effectively uncovered tacit procedural knowledge, an outcome consistent with theories of collaborative emergence (Sawyer, 2006, 2014, 2018).

Furthermore, the FTA-guided follow-up interviews in April and May markedly increased the externalization of purpose-based knowledge, with the per-interview average count rising to approximately four times the pre-FTA average. FTA’s use of backward reasoning from observed outcomes facilitated a more profound articulation of the rationales behind expert actions.

The workshop fostered the emergent externalization of tacit knowledge through group collaboration, while the FTA-guided follow-up interviews proved superior for articulating context-sensitive, purpose-based knowledge.

### 4.2 KG findings

**Figure 4:**
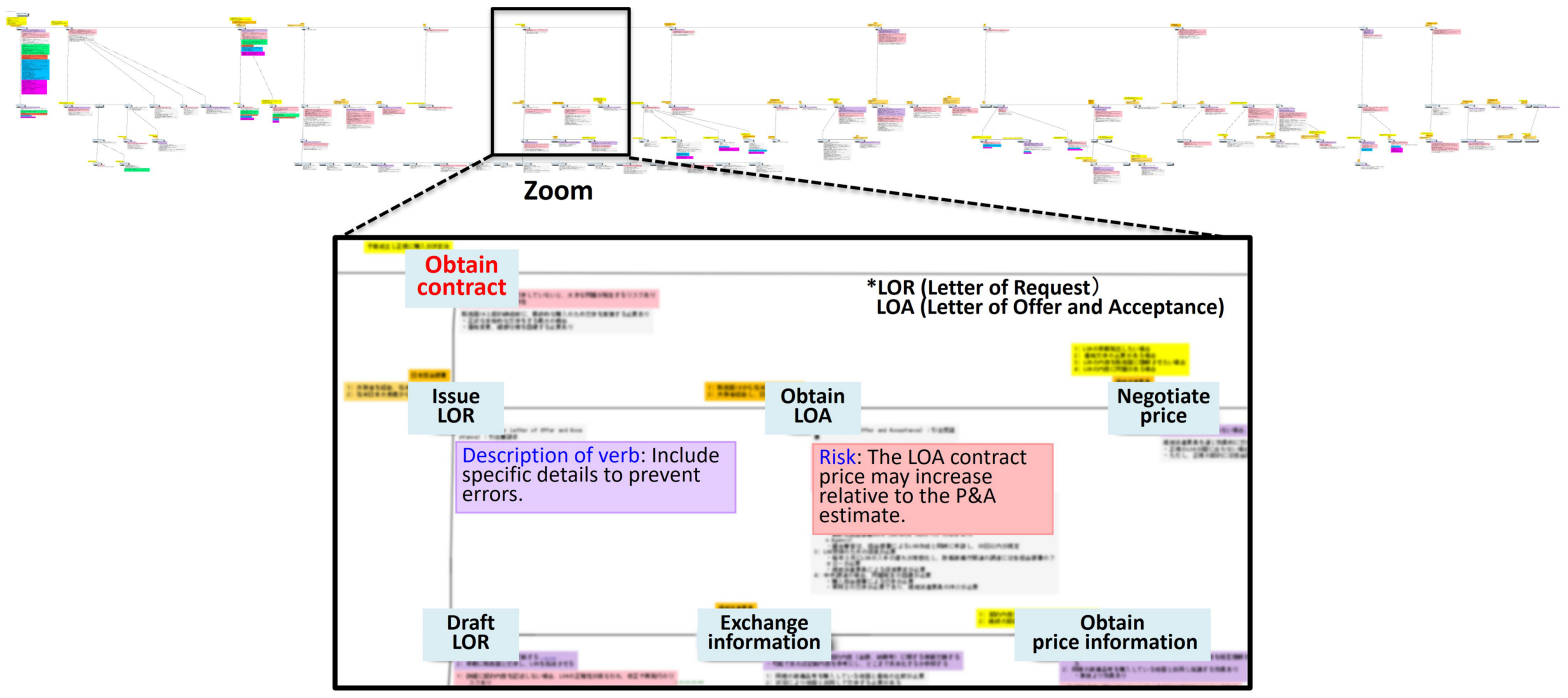
Procedure-based KG.

**Figure 5:**
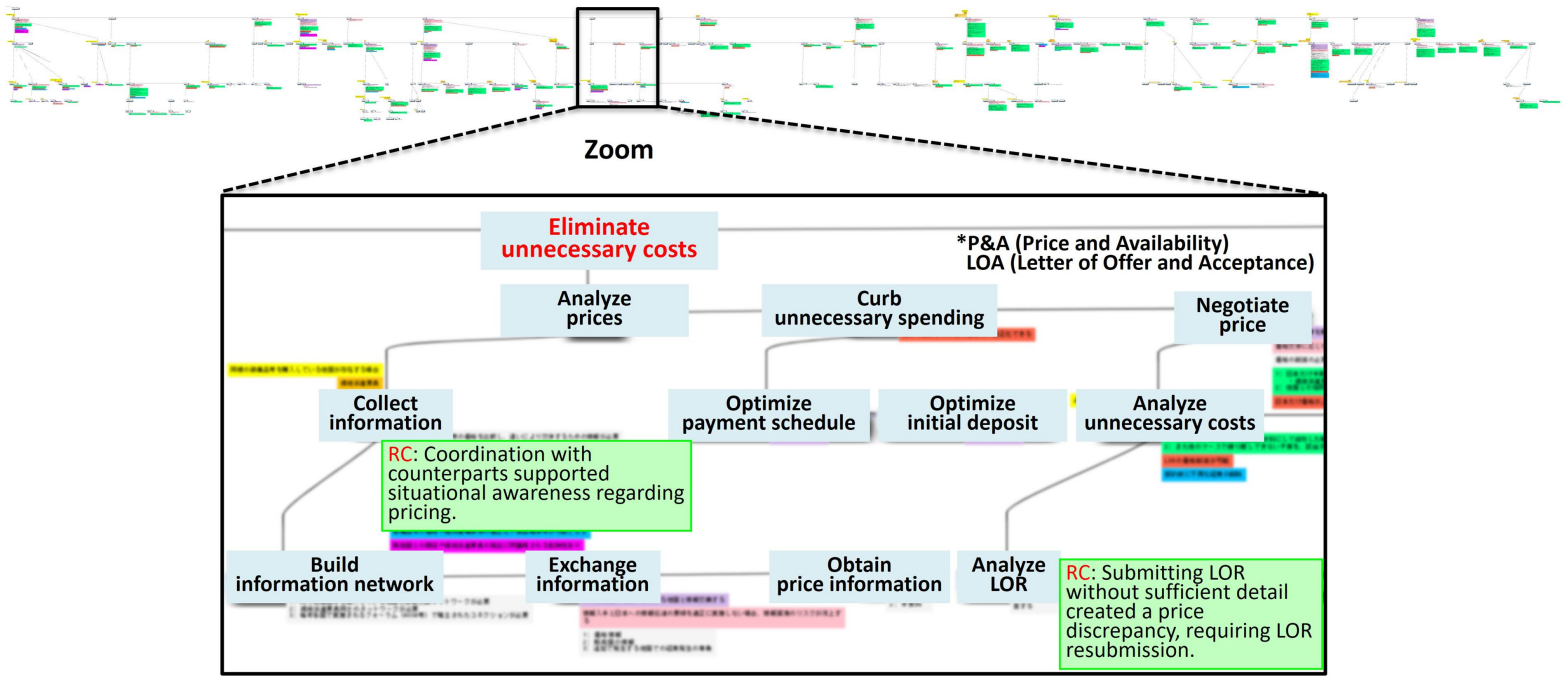
Purpose-based KG.

We applied our structured methodology to the FMS procurement process to organize and externalize expert knowledge. Based on data from interviews and workshops, we used a purpose-driven modeling approach where each procedure was hierarchically decomposed to ensure both clarity and reusability. The resulting KGs are presented in Figures 4, 5.

Figure 4 displays the procedure-based KG, which structures the procedure-based knowledge for the FMS case. The top-level action node, “Conduct FMS procurement,” is decomposed into 12 child nodes representing the major phases of the process, such as “Verify FMS procurement effectiveness,” “Select FMS procurement,” “Collect information,” “Get quote,” “Request budget,” “Obtain contract,” “Execute contract,” “Coordinate transportation,” “Receive delivered items,” “Maintain items,” “Dispose items,” and “Close out contract.”

Figure 5 shows the purpose-based KG, which structures the corresponding purpose-based knowledge. The top-level purpose node, “Conduct FMS procurement,” is decomposed into 17 child nodes that capture the strategic goals of the process, such as “Procure cutting-edge deliverables,” “Procure reliable deliverables,” “Adapt Japanese specifications,” “Resolve version-upgrade issues,” “Prevent errors,” “Prevent price increases,” “Eliminate unnecessary costs,” “Prevent contract delays,” “Avoid accounting-system differences,” “Prevent delivery delays,” “Resolve transportation issues,” “Resolve maintenance issues,” “Resolve defects,” “Ensure smooth disposal,” “Achieve early contract closeout,” “Achieve efficient procurement,” and “Conduct equal-footing negotiations.”

The two complementary knowledge types are integrated into two linked graphs connected by explicit purpose-action links (Ijuin and Nishimura, 2023). Some actions map to a single purpose, whereas others map to multiple purposes, representing not only what is done but also why it is done. Following the method described in Section 3, relevant nodes and edges are annotated to ensure that causal reasoning and provenance remain auditable across both graphs.

**Figure 6:**
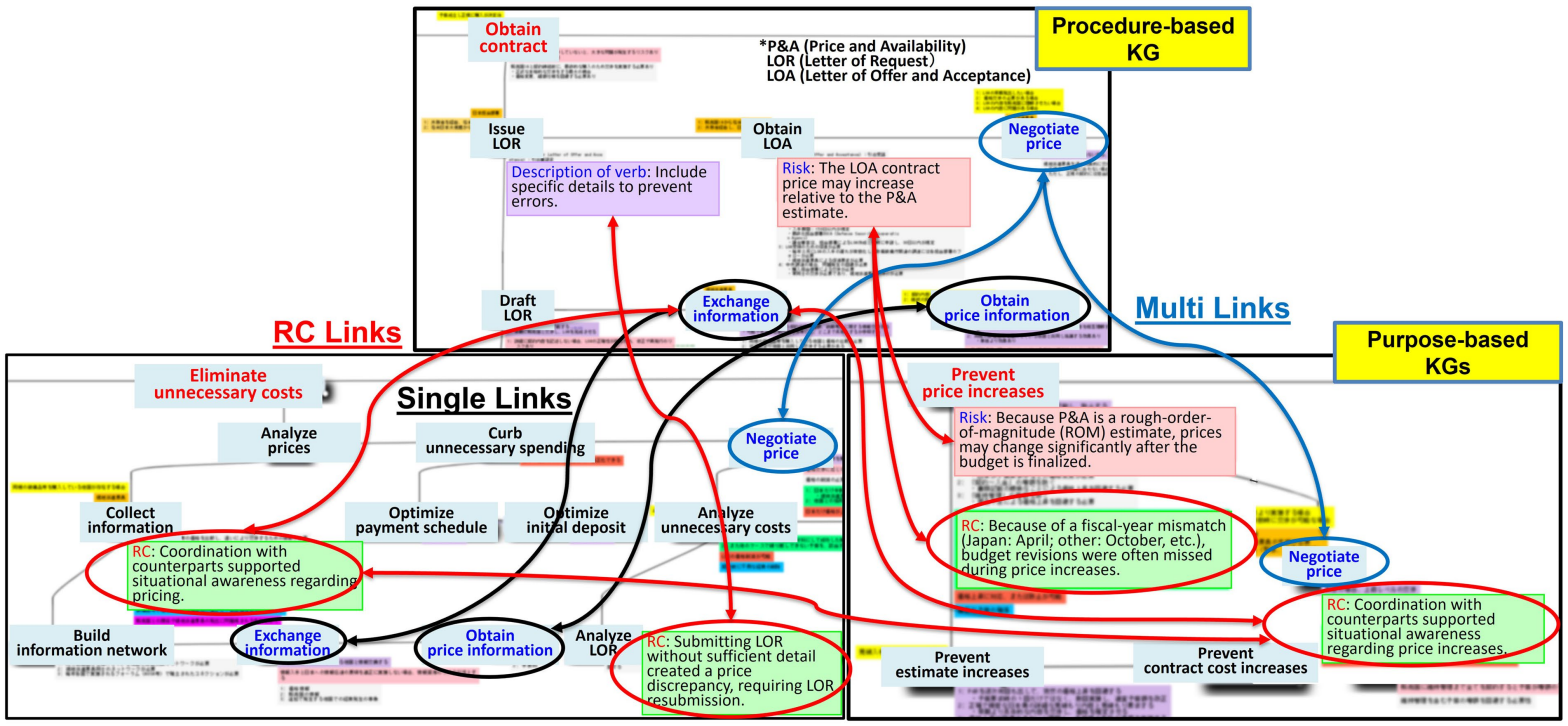
Example of links between the procedure- and purpose-based KGs.

Figure 6 provides an example of this cross-graph linkage, illustrating how an action such as “Obtain contract” is connected to purposes such as “Eliminate unnecessary costs” and “Prevent price increases.” Some actions (e.g., “Exchange information” and “Obtain price information”) are associated with a single purpose (indicated by black arrows and circles), whereas others (e.g., “Negotiate price”) are linked to multiple purposes (indicated by blue arrows and circles), revealing both the task flow and its underlying rationales.

A key novelty of this study, also illustrated in Figure 6, is the representation of FTA-derived RCs. The RCs are shown as green boxes, with specific actions identified to avoid failure modes. A single RC can link to multiple purposes in the purpose-based KG and to multiple action nodes in the procedure-based KG. In Figure 6, the RC connects to contextual elements such as the verb description and risk boxes, forming a coherent structure that supports governance and reuse. This structuring is designed to make tacit knowledge more visible and reusable in complex domains such as international R&D and procurement, and it may support transfer under institutional and temporal–spatial constraints by providing auditable provenance and cross-case retrievability.

### 4.3 Quantitative analysis of RCs

This section quantifies the number of case-attached actions generated per RC within the FTA-adapted CHARM process (Section 3). Using the proposed notation, each RC is represented by purpose-action pairs with clear provenance, which are embedded as typed links within the two KGs. Following the initial interviews, FTA-guided sessions were used to identify avoidance behaviors and candidate actions based on observed outcomes; these were then encoded as purpose-action pairs and registered under each RC. By construction, the counts are zero-truncated, as an RC is created only when at least one action is elicited.

This process resulted in 133 RC-linked knowledge units—recorded as case-attached actions—anchored by 42 RCs (*n* = 42). These units are embedded within the KGs as explicit, provenance-bearing links that strengthen the case-based validation of purposes and improve traceability while keeping CHARM’s goal-means semantics intact. Therefore, the RC mechanism does more than store narratives; it systematically increases the reusable links between purposes and actions, providing auditable provenance. In our data, a typical RC contributes 1 to 4 case-attached actions (median = 2), which meaningfully increases connectivity without altering CHARM’s semantics.

As summarized in Table 4, a typical RC contributes between 1 and 4 actions; median = 2, inter-quartile range (IQR) = 2–4, range: 1–12, standard deviation (SD) = 2.34, and mean *(M)* = 3.17 (95% CI: 2.44, 3.89). The distribution is right-skewed, with most RCs including 1 to 4 actions, whereas a few have higher counts (7, 9, and 12). These links enhance the validation of purposes and improve traceability across the two graphs. For transparency with this count data, we prioritize nonparametric summaries (median, IQR) and report a 95% CI for the mean; no imputation was applied, and all RCs were equally weighted. A 95% bootstrap CI for the mean was computed (a t-based CI yielded similar bounds); no imputation was applied, and all RCs were equally weighted.

**Table 4:** Summary statistics for case-attached actions per RC (*n* = 42).

*n*	Total	Mean	95% CI	Median	IQR	Range	SD
42	133	3.17	2.44–3.89	2.0	2–4	1–12	2.34

**Table 5:** Representative practitioner feedback.

ID	Excerpt	Interpretation focus
Q1	“Know-how and related knowledge had been dispersed and remained person-dependent; being able to transfer it in a structured form is perceived as effective.” (Practitioner, de-identified; overseas work experience)	Institutionalization; reducing person-dependence; continuity across handovers
Q2	“Because baseline knowledge and skills vary among assignees, I strongly want this type of knowledge to be systematically shared and taught.” (Practitioner, de-identified; supervisory experience)	Training needs; accessibility for novices; perceived usefulness for transfer

Note: de-identified; raw interview evidence.

### 4.4 Qualitative validation based on practitioner feedback

To complement the above quantitative indicators, we report de-identified practitioner feedback on the perceived usefulness of the structured knowledge artifacts for handover-oriented reuse and institutionalization (Table 5).

After the initial structuring, we conducted validation interviews with ten practitioners involved in the post-structuring activities. The excerpts below, reported as raw interview evidence (de-identified), highlight two recurrent themes: (i) mitigating person-dependent know-how and knowledge dispersion and (ii) supporting transfer under heterogeneous baseline skills among assignees.

## 5 Discussion

This section discusses the implications of applying the FTA-adapted CHARM approach to knowledge transfer in Japan’s international security cooperation for R&D and procurement. We interpret how the workshop and FTA-guided follow-ups contributed differently to procedure- and purpose-based knowledge externalization, and we consider the role of RC anchoring for reuse across cases.

### 5.1 Synthesis of key findings

The results indicate a complementary output profile across elicitation methods. The facilitated workshop yielded the largest gains in procedure-based knowledge, consistent with the view that interactional co-creation can surface procedural details beyond individual recall (Sawyer, 2006, 2014, 2018). By contrast, the FTA-guided follow-up interviews markedly increased the externalization of purpose-based knowledge, suggesting that backward reasoning from observable outcomes is effective for eliciting conditional purposes and associated decision rules that standard interviews tend to leave implicit.

Practitioner feedback (Section 4.4) further indicates perceived usefulness of the structured artifacts for handover-oriented reuse, particularly in relation to mitigating person-dependent know-how and supporting transfer where assignees’ baseline skills vary.

### 5.2 Interpretation and positioning relative to existing approaches

#### 5.2.1 Complementary roles of workshops and FTA in knowledge structuring

The workshop functioned as a co-creative device that surfaced procedural details and externalized expert knowledge as participants jointly validated action flows, harmonized terminology, clarified pre- and post-conditions and information dependencies, and validated shared procedures. Consistent with Sawyer’s work on emergent collaboration, new knowledge arose from interactional chains rather than from individual recall; in our data, the workshop produced the largest monthly gains in procedure-based knowledge (Sawyer, 2006, 2014, 2018). These interactions also helped stabilize procedure descriptions for reuse across handovers.

FTA complemented this by providing a deductive elicitation method. By starting from observable outcomes (e.g., delivery delays, price increases) and reasoning backward, this approach surfaced conditional purposes, decision rules, and latent failure paths that standard interviews struggled to reveal. This complementarity is consistent with the observed differences in elicited procedure- and purpose-based knowledge.

This finding aligns with research showing that deeply internalized knowledge resists direct narration and benefits from structured scaffolds (Polanyi, 1966; Nonaka and Takeuchi, 1995). The reverse-causal questioning in FTA helped participants articulate embedded rationales that typically remain pre-reflective, consistent with Polanyi’s account of tacit knowledge and Nonaka and Takeuchi’s concept of internalization.

#### 5.2.2 Role of RC-anchored notation

**Figure 7:**
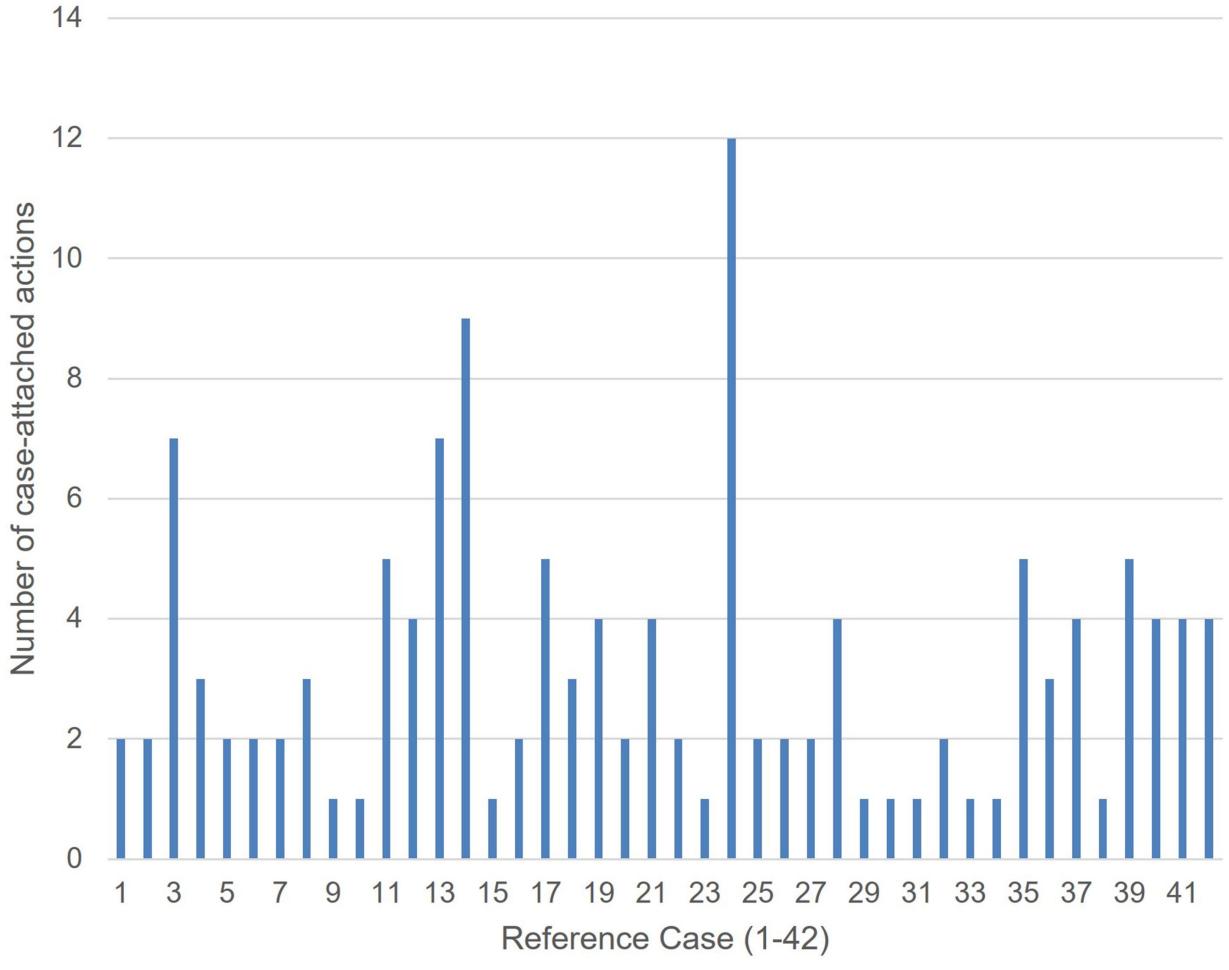
Distribution of case-attached actions across the 42 RCs.

**Figure 8:**
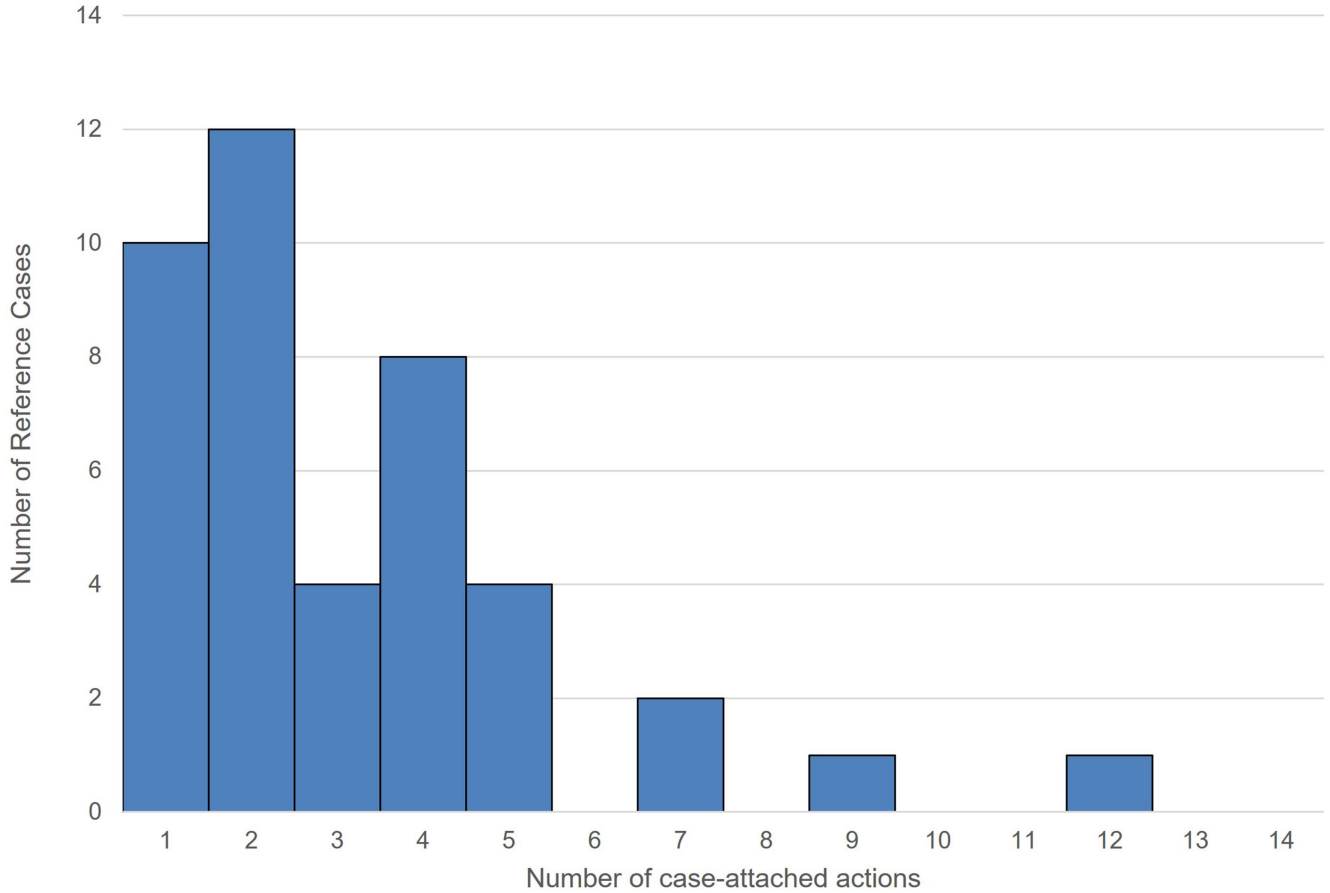
Histogram of case-attached action counts per RC (*n* = 42; *bin width* = 1).

As each purpose-action link is anchored to an RC, the notation supports cross-case counting and prioritization rather than remaining purely descriptive. In our dataset, the 133 RC-linked units from 42 RCs exhibit a zero-truncated count structure, which enables subtheme-level analysis for implementation planning. The distribution is right-skewed: a typical RC contributes 1 to 4 case-attached actions (*M* = 3.17; *95% CI*: 2.44–3.89; *IQR* = 2–4), indicating concentrated but uneven leverage across cases (Figures 7, 8).

Moreover, anchoring links in RCs provides background and situational cues that a bare structural representation cannot capture. While no scheme can exhaustively cover tacit knowledge, RCs offer practical hints that aid understanding and guide further elicitation. The RC mechanism also enables provenance-bearing purpose-action links to be queried and reviewed across cases (e.g., by purpose name, process step, or RC identifier), while preserving the goal-means semantics of the two KGs.

#### 5.2.3 Statistical characterization of priority counts

Using the frequency of RC-tagged case-attached actions (Figure 8), we identified four high-priority subthemes from the data (count ≥ 7):

1. Information collection and negotiation support (12)
2. Fiscal-year mismatches between Japan and the partner country (9)
3. Timing of price negotiation (7)
4. Price escalation mitigation (7)

Focusing implementation on these subthemes can support concrete problem-solving roadmaps (e.g., aligning budgeting calendars, planning early information capture, scheduling negotiation windows, and preventing price escalation) and provides clear targets for monitoring future cycles.

#### 5.2.4 Comparative advantages and boundary conditions

Relative to interview-only capture, the proposed approach adds two scaffolds: co-creative stabilization of procedures (workshop) and deductive articulation of rationales (FTA). Relative to CHARM-only structuring, it strengthens the explicitness of purpose-based reasoning by using outcome-driven backward questioning and by registering RC-anchored purpose-action units as typed links. Its contribution relative to conventional FTA is not quantitative risk modeling but a qualitative elicitation and articulation use that is integrated into CHARM-consistent representations and reused across cases through RC identifiers.

These comparative advantages are most salient under boundary conditions common in international security cooperation, such as temporal gaps between projects, personnel rotation, and constraints on direct handover. Conversely, the approach may be less informative when (i) outcomes cannot be clearly defined as “top events,” (ii) expert access is severely limited, or (iii) organizational constraints restrict what can be externalized.

### 5.3 limitations

The findings of this study should be interpreted in light of several limitations. First, the evaluation centered on a single national context (Japan) and programmatic case (FMS). Hence, generalization to other frameworks (e.g., international joint R&D or DCS) or countries requires further investigations.

Second, the study employed a qualitative, expert-elicitation design (*n* = 30 participants). While this sample size is suitable for surfacing tacit knowledge in a specialized domain, it is not intended for estimating population parameters. As frontline teams are typically small in this operational setting (few practitioners per year), our sample represents a substantial share of the practitioner population over recent cycles. However, the findings may have been affected by selection and recall biases.

Third, the study relies on expert elicitation and workshop interaction. Participant composition, facilitation dynamics, and researchers’ interpretive decisions may have influenced what is externalized and how it is framed, even when terminology harmonization and cross-checking are performed.

Fourth, parts of the elicited knowledge are retrospective accounts. Recall limitations may have affected completeness and the salience of failure/avoidance narratives, potentially shaping RC anchoring and the resulting counts.

Fifth, in policy- and security-sensitive settings, restrictions on what can be externalized, stored, or shared limit both the granularity of reported evidence and the reproducibility of full case materials. This also constrains cross-organizational comparison.

Sixth, our adaptation of FTA is qualitative and does not account for cost or schedule risk impacts. In addition, constructing and maintaining the mechanism entails operational costs for authoring, curation, and maintenance (e.g., terminology management, versioning, and access control).

### 5.4 Future work

Building on the findings of this study, future work should focus on the following three areas:

First, scope extension and portability testing are needed. This involves extending the evaluation beyond FMS to multi-program, multi-site, and cross-national settings under diverse institutional and classification regimes. Such work could also analyze how policy-intensive contexts shape knowledge transfer. Concurrently, the data assets should be prepared for deployment in generative-AI assistants that can operate on structured purpose-action models while retaining traceability and interpretability. Comparative evaluation against plausible baselines (e.g., interview-only capture, CHARM-only structuring, or conventional FTA documentation) should also be conducted using shared criteria such as traceability, coverage of purposes, and handover-oriented reuse.

Second, mechanisms for sustained use should be developed. This includes designing an organizational process for continuous knowledge externalization and accumulation, incorporating version control and access protocols, and instituting dashboards for monitoring RC-anchored counts. Appointing knowledge managers and engaging experienced practitioners will be crucial for both externalization and internalization. By utilizing provenance-bearing purpose-action structures, these steps will help preserve crucial knowledge across temporal–spatial discontinuities and integrate individual expertise into a shared organizational knowledge base.

Third, practitioner enablement and tool integration are critical. This entails publishing operational playbooks for workshops and FTA follow-ups, training facilitators, and integrating KG outputs into routine tools (e.g., checklists and review prompts). Where appropriate, human-AI support can be explored, provided that provenance and interpretability of RC-anchored links are preserved.

## 6 Conclusion

This study examined sustainable knowledge transfer in Japan’s international security cooperation for R&D and procurement, where personnel rotation, temporal–spatial discontinuities, and disclosure constraints hinder direct handover of practitioner expertise. To address this problem, we proposed an RC-anchored FTA-adapted knowledge structuring workflow that couples CHARM with qualitative FTA as a deductive elicitation and articulation scaffold, producing two auditable, linked KGs (the procedure- and purpose-based knowledge graphs) connected by RC-tagged purpose-action links.

Our contributions are threefold. Methodologically, integrating FTA and RC tagging into CHARM provides an explicit route from observable outcomes (“top events”) to provenance-bearing purpose-action units, strengthening auditability and procedure-level analytic reproducibility. Theoretically, the findings clarify complementary roles across elicitation settings: workshops help stabilize shared procedures, whereas FTA-guided backward reasoning elicits conditional purposes and decision rules that often remain implicit in standard interviews. Practically, the resulting artifacts (42 RCs and 133 case-attached actions) make many-to-many purpose-action relationships queryable and enable prioritization of high-frequency subthemes to support implementation planning and handover-oriented reuse.

Overall, the resulting artifacts are designed to sustain continuity during constrained handovers, connect individual expertise with organizational sharing, and provide structured data for purpose-aware AI assistants. Ultimately, the RC-anchored purpose-action framework offers a reusable, auditable, and machine-interpretable basis for sustaining knowledge transfer in this high-stakes environment. By making the “why” of expert practice as explicit and reusable as the “what,” our framework is intended to reinforce continuity, sharpens prioritization, and provides a practical foundation for human-AI collaboration under real-world, policy-intensive constraints.

## Data Availability

The original contributions presented in the study are included in the article/supplementary material, further inquiries can be directed to the corresponding author.
