# Correction: Fault tree analysis-adapted knowledge structuring: a case study of sustainable international security cooperation

**DOI:** 10.3389/frai.2026.1808388

**Published:** 2026-02-23

**Authors:** Frontiers Production Office

**Affiliations:** Frontiers Media SA, Lausanne, Switzerland

**Keywords:** knowledge structuring, fault tree analysis, CHARM, knowledge graph, tacit knowledge, sustainable knowledge transfer, international security cooperation, R&D and procurement

The reference for Government of Japan, **2022** was erroneously written as Government of Japan, **2002**. It should be Government of Japan, **2022**.

The original version of this article has been updated.

